# Supplementary material for: All-at-once RNA folding with 3D motif prediction framed by evolutionary information
Source: Nat Methods. 2025 Oct 3;22(10):2094–106. doi: 10.1038/s41592-025-02833-w (PMC12510878; doi:10.1038/s41592-025-02833-w)
Supplement: Supplementary file 3 — Source code to run CaCoFold-R3D. Data and results. [file 41592_2025_2833_MOESM3_ESM.gz › supplemental_material/results/CaCoFold-R3D/CaCoFold-R3D_Rfam/RF02474_GlsR5.R2R.sto.pdf]

RF02474_GlsR5

5′ -○○ U U A A A A G C U G U G A U G A C A G G U U C U U G C C C C G U A U G A C C C U G C G A U G A G U U A U A C A A A A G A A C G C A Y Y C A A G C C A A C C G R C U G A G C U C
